# Facial nodules and hypopigmented plaques of the body

**DOI:** 10.1016/j.jdcr.2025.10.037

**Published:** 2025-10-28

**Authors:** Ryan Gall, Catherine Brahe

**Affiliations:** a Department of Dermatology, Naval Medical Center San Diego, San Diego, California; b Department of Dermatology, Naval Medical Center Camp Lejeune, Camp Lejeune, North Carolina

**Keywords:** Hansen’s disease, leonine facies, lepromatous leprosy, leprosy, multibacillary

## Case description

**Fig 1:**
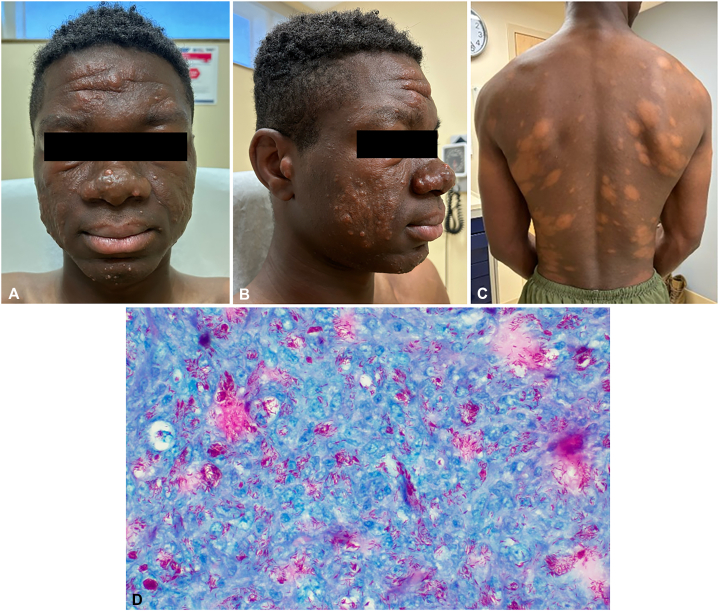
Facial nodules, edema, and “leonine facies” **(A, B)**. Hypopigmented annular plaques of the trunk **(C)**. Globi seen on AFB Fite stain **(D)**.

A 24-year-old male presented to dermatology with 6 months of progressive facial changes. Extensive scattered facial granulomatous skin-colored papules and notable edema were observed (Fig 1, *A* and *B*). Further examination showed scattered hypopigmented annular and indurated plaques of the trunk and upper thighs (Fig 1, *C*). The patient reported no preceding illnesses or recent travel, no constitutional symptoms, and no close contact with similar symptoms. The patient immigrated to the United States from Cameroon with his family approximately 6 years prior to the onset of symptoms.

**Question: What is the most likely diagnosis?**

**A.** Systemic amyloidosis
**B.** Scleromyxedema
**C.** Cutaneous sarcoidosis
**D.** Hansen’s disease (leprosy)
**E.** Mycosis fungoides

Correct answer: **D.** Hansen’s disease (leprosy)

## Discussion

A punch biopsy demonstrated granulomas of foamy histiocytes with perineural involvement, a Grenz zone, and innumerable acid-fast bacilli (globi) identified on Fite stain (Fig 1, *D*). A diagnosis of lepromatous leprosy/multibacillary Hansen’s disease was made. Leprosy or Hansen’s disease is a chronic infection caused by *M. leprae* or *M. lepromatosis*. Hansen’s disease is considered a neglected tropical disease, with more than 200,000 new cases reported annually.^1^ The average incubation period is approximately 5 years, though incubation can range from 2 to 30 years.^1^ Lepromatous leprosy is the most severe presentation of multibacillary leprosy, characterized by minimal cell-mediated immune response and a high humoral response. Patients with lepromatous leprosy often present with extensive, symmetric skin involvement, which may be hypopigmented or erythematous. Facial involvement may cause diffuse thickening of the dermis, leading to a characteristic “leonine facies.” Vision loss and a saddle nose deformity may also result from uncontrolled disease. Prolonged close contact is required for human-to-human spread of the disease, and patients are considered to be noninfectious 72 hours after their first treatment dose.^1^ Approximately 95% of the general population are thought to be naturally immune to leprosy and will not contract the disease even with long-term exposure.^1^ The patient was suspected to have contracted Hansen’s disease prior to immigrating to the United States, though the specific source of exposure was not identified.

Diagnosis requires clinical recognition of symptoms, though testing such as skin biopsy, slit skin smears, polymerase chain reaction, and nerve conduction studies can all aid in a diagnosis of Hansen’s disease.^2^ The causal organisms can be highlighted in tissue with acid-fast bacilli stains, though paucibacillary or tuberculoid cases may show few, if any, identifiable organisms.^2^ The World Health Organization recommends specific multidrug therapy regimens based on whether the patient has paucibacillary or multibacillary disease.^1^ Immune reactions, such as type 1 (reversal reactions) or type 2 (erythema nodosum leprosum), occur frequently either before or after initiation of treatment. Immune reactions are considered a medical emergency; prompt recognition and treatment with agents such as corticosteroids or thalidomide are critical to prevent severe morbidity.^1^

The patient was initiated on a 24-month course of multidrug therapy consisting of rifampin 600 mg monthly, moxifloxacin 400 mg monthly, and minocycline 100 mg daily. The patient had substantial improvement in cutaneous symptoms after 1 month of treatment.

## Conflicts of interest

None disclosed.
